# Street Collections

**Published:** 1896-07-11

**Authors:** 


### The Hospital

An Institutional Journal of

The Medical Sciences and Hospital Administration, with

AN EXTRA NURSING SUPPLEMENT.

Vol. XX.—No. 511.] [July 11, 1896.

### Street Collections.

The evils and dangers of street collections in London increase daily. The hospitals of London have suffered by the street collections made on one day in the year throughout London in connection with the Hospital Saturday Fund. They have suffered because these collections irritate very many people who give largely to hospitals, and so probably lead to the withholding of much larger sums than the total amount received through their agency. They are further objectionable because they entail duties upon a large number of women who—unconsciously we hope—injure themselves by touting in this way for any cause, however worthy in itself. In a few provincial towns street collections on one day in the year have been tolerated as an advertisement for the Hospital Saturday movement; but the most thoughtful observers are of opinion that, even as an advertisement, street collections in practice do as much harm as good. The working classes, to whom the Hospital Saturday Fund particularly appeals, are wisely jealous of any outside interference, and the most worthy members of that class realise that there is a loss of dignity about street collections, and that they lay the Fund open to the criticism that the contributions received are not, after all, working men's gifts pure and simple.

Unfortunately, the bad example set by the Hospital Saturday Fund in London has been followed by the Lifeboat Institution, to the injury of that important national charity, though the harm resulting has not at present made itself felt to the extent it is likely to do by the loss of legacies, and by a diminished income from voluntary contributions, and especially from annual subscriptions. We are glad to learn that the direct benefits derived by the Lifeboat Institution from the street collections are relatively unsatisfactory, and we can only hope that those wise members of its council who are strongly opposed to this evil system will reopen the subject by summoning a special meeting and reverse the decision of the narrow majority by which the proposal to institute street collections in connection with the Lifeboat Saturday movement was, we understand, originally carried. Unless such a step be speedily taken we are confident, from what we hear on all hands, that the income of the Lifeboat Institution is likely to suffer materially, and its usefulness to be much crippled.

There is nothing so infectious as evil example; and the condition of the public streets, which are becoming thronged with collectors for all sorts of objects, proves this contention to be true. Very few reputable charities, except the two already alluded to, have heretofore resorted to the system, but a number of one-man institutions are pushing street collections with an assiduity and persistence which demands the prompt action of the County Council. London appears to be threatened with a street collection not only upon every Saturday, but possibly upon every day in the year—collections not organised by representative committees of citizens, nor necessarily even by the committees of particular institutions, but, as the proceedings in the police courts have proved over and over again, mere attempts upon the part of needy beggars to raise the wind by masquerading in the clothes of the reputable institutions. Street collections are carried on largely in all parts of London, and the collectors are paid at least 2s. 6d. a day each by the persons who take possession of the funds thus abstracted from the pockets of the public. We hope that every possible pressure will be brought to bear upon the Home Secretary, and that the members of the London County Council will take up the matter and combine to secure the passing of a bye-law prohibiting street collections in London similar to the bye-laws which, we understand, have proved advantageous in provincial towns.

It is well known that the most influential and best-informed members of the Metropolitan Hospital Saturday Fund Council are opposed to the street collections, and would gladly give them up. How strong is this feeling may be gleaned from the circumstance that this year for the first time the intimation that street collections would take place was signed not by the chairman or the treasurer of the council, but by the secretary of the Saturday Fund. Seeing the evils which have arisen from the institution of street collections in London by the Hospital Saturday Fund, and the undoubted injury which they cause to the whole of the voluntary hospitals, the time has nearly arrived, if it has not already come, when it may be necessary for the managers of these institutions to take combined action with a view to preventing their continuance. Next year, if the Hospital Saturday Fund persist in holding street collections in London, we hope that the more influential of the hospital authorities will address a letter to the public papers immediately before the Saturday in July on which these collections are held, asking the public to refrain from giving any money to the collectors who may be found in the streets on that day. The amount raised by them for the Hospital Saturday Fund is relatively so small as to be inappreciable when the whole expenditure of the hospitals is taken into consideration, and we earnestly hope that the wiser heads connected with the Council of the Hospital Saturday Fund will combine to carry a resolution giving up these collections. If this step be taken everybody will rejoice, and the streets of London will be freed from a nuisance which every year makes more and more intolerable.

